# Laboratory assessment of anti-thrombotic therapy in heart failure, atrial fibrillation and coronary artery disease: insights using thrombelastography and a micro-titre plate assay of thrombogenesis and fibrinolysis

**DOI:** 10.1007/s11239-016-1344-5

**Published:** 2016-03-04

**Authors:** Y. C. Lau, Q. Xiong, P. Ranjit, G. Y. H. Lip, A. D. Blann

**Affiliations:** University of Birmingham Institute for Cardiovascular Sciences City Hospital, Dudley Road, Birmingham, B18 7QH UK; Cardiovascular Department, The Second Affiliated Hospital of Nanchang University, Nanchang, China

**Keywords:** Thrombelastograph, Thrombosis, Fibrinolysis, Haemostasis

## Abstract

As heart failure, coronary artery disease and atrial fibrillation all bring a risk of thrombosis, anti-thrombotic therapy is recommended. Despite such treatment, major cardiovascular events such as myocardial infarction and stroke still occur, implying inadequate suppression of thrombus formation. Accordingly, identification of patients whose haemostasis remains unimpaired by treatment is valuable. We compared indices for assessing thrombogenesis and fibrinolysis by two different techniques in patients on different anti-thrombotic agents, i.e. aspirin or warfarin. We determined fibrin clot formation and fibrinolysis by a microplate assay and thromboelastography, and platelet marker soluble P selectin in 181 patients with acute or chronic heart failure, coronary artery disease who were taking either aspirin or warfarin. Five thromboelastograph indices and four microplate assay indices were different on aspirin versus warfarin (p < 0.05). In multivariate regression analysis, only microplate assay indices rate of clot formation and rate of clot dissolution were independently related to aspirin or warfarin use (p ≤ 0.001). Five microplate assay indices, but no thrombelastograph index, were different (p < 0.001) in aspirin users. Three microplate assay indices were different (p ≤ 0.002) in warfarin users. The microplate assay indices of lag time and rate of clot formation were abnormal in chronic heart failure patients on aspirin, suggesting increased risk of thrombosis despite anti-platelet use. Soluble P selectin was lower in patients on aspirin (p = 0.0175) but failed to correlate with any other index of haemostasis. The microplate assay shows promise as a tool for dissecting thrombogenesis and fibrinolysis in cardiovascular disease, and the impact of antithrombotic therapy. Prospective studies are required to determine a role in predicting thrombotic risk.

## Introduction

The leading cause of mortality and morbidity in the developed world is cardiovascular disease. Three of the major manifestation of this disease are heart failure, whether presenting acutely or chronically, coronary artery disease, and atrial fibrillation. Each of these conditions may be present alone, or in concert, as they all share common risk factors such as atherosclerosis, hypertension, diabetes or chronic kidney disease. Furthermore, the development of any one of these conditions is itself a precursor for future development of the other two diseases. These three conditions are also linked the development of other thromboembolic disease such as ischaemic stroke and systemic thromboembolism (notable deep vein thrombosis and pulmonary embolism) [1–4].

A key treatment of cardiovascular diseases is anti-thrombotic therapy with antiplatelet agents such as aspirin and clopidogrel, and with oral anticoagulants such as the vitamin K antagonist (VKA) warfarin and non-VKA oral anticoagulants (NOACs) [5–8]. However, although these strategies are effective, there remains a considerable risk of additional thrombosis in patients on these drugs despite best clinical practice. One strategy to improve clinical outcome is to provide personalised thrombotic risk assessment by assessing patients with or at increased risk of thrombosis and then target those at highest clinical risk, and/or identifying those in whom standardised antithrombotic therapy seems ineffective. In both these respects, the laboratory can provide direct evidence of an increased risk of thrombosis and the efficacy of anti-thrombotic drugs, the international normalised ratio (INR) being the best example, although this test is not optimum for assessing NOACs [9–11]. However, although the INR provides essential information on the effects of VKAs, it has limited scope, leaving room for the development of other tests.

Whole blood and fibrin clot formation and lysis may also be studied in vitro with devices such as the thrombelastograph, and in a micro-titre plate assay, of which there are several variants [12–14]. We have recently described and validated an adaptation of the latter method for testing haemostasis in vitro with a micro-plate assay, and have compared it to the thrombelastograph [15]. The thrombelastograph has been used clinically in examining the effects of warfarin, heparin and the NOACs [16–19].

We hypothesised that the microplate assay is better able to discriminate those patients with cardiovascular disease (heart failure, atrial fibrillation, coronary artery disease) on different antithrombotic treatments than is the thromboelastograph. The standard antithrombotic treatment for atrial fibrillation is oral anticoagulation (almost always warfarin), which brings a disrupted coagulation system. Conversely, coronary artery disease patients on aspirin should have minimum alteration to their coagulation system, whereas acute heart failure and chronic heart failure patients may be on either treatment [7]. This allows comparisons of the microplate assay with the thromboelastograph in subjects on different antithrombotic therapies. To provide a perspective of acute compared to chronic, stable disease, we recruited a small number of in-patients with acute heart failure, and to perspective of platelet function and haemostasis between those on aspirin and those on warfarin, we measured levels of platelet marker soluble P selectin, known to be increased in cardiovascular disease [20].

## Subjects, materials and methods

### Subjects

Venous blood was obtained from 57 patients with chronic heart failure, 20 patients with acute heart failure, 44 patients with coronary artery disease but no heart failure or atrial fibrillation, and 60 patients with atrial fibrillation but no heart failure or coronary artery disease, all of whom were attending hospital as in-patients or as out-patients. Exclusion criteria were aged <18 years, had active or recent (<12 months) malignancy, active immunological disease, pregnancy, chronic liver disease, recent or chronic infections, chronic inflammatory disease, connective tissue disease, recent stroke/acute coronary syndrome (within 2 months), active bleeding, recent arterial/venous thrombosis or recent surgery, known haemophilia or thrombophilia (such as Factor V Leiden, Protein C/S/antithrombin deficiency, antiphospholipid syndrome), use of an anti-platelet other than aspirin, use of a VKA other than warfarin, or unable to give consent. Heart failure was defined by recent, routine echocardiography within past 6 months and with a documented severe left ventricular systolic dysfunction with ejection fraction ≤35 %, atrial fibrillation by typical changes on routine 12-lead electrocardiogram, and coronary artery disease by previous myocardial infarction (>12 months), coronary artery bypass grafting, or >50 % stenosis of a least one coronary artery defined by coronary angiogram. Standard clinical, laboratory, and demographic data were collected (Table 1). Local research ethics committee approval was obtained and all participants gave written informed consent.

**Table 1:** Thromboelastograph and microplate assay indices

	Process assessing
Thrombelastograph indices	
R time	Time from the initiation of the test until the point where the clot begins to form
K time	Interval from the split point of the test to the point where the fibrin cross-linking provides enough clot resistance to produce a 20-mm amplitude
Angle	Angle formed by the slope of a tangent line traced from the R time to the K time: reflects the rate at which the clot forms
Maximum amplitude	Maximum amplitude of the clot dynamics, reflecting clot strength
LY30	Percentage of the clot that has lysed 30 min after the time of the maximum amplitude
Microplate assay indices	
Lag time	Time from the initiation of the test to the point where the clot begins to form
Rate of clot formation	Change in optical density over time from the beginning of clot formation (i.e. the end of the lag time) to the point of maximum optical density
Maximum optical density	Maximum optical density, reflecting clot thickness
Rate of clot dissolution	Reduction in optical density from maximum to the plateau phase, divided by the time between the two points
T50	Time for 50 % of the clot to lyse

**Fig. 1:**
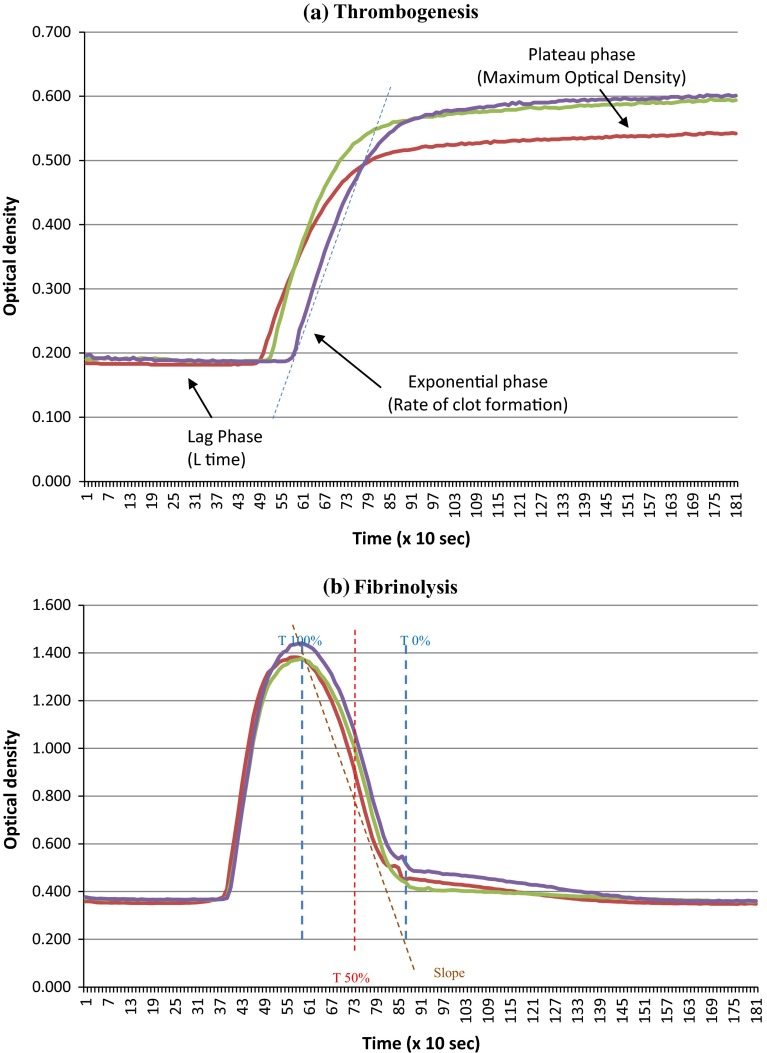
The Microplate assay. **a** Thrombogenesis. The plot shows changes in optical density as the fibrin clot forms. Triplicate plots are shown. **b** Fibrinolysis. The plot shows changes in optical density as the fibrin clot forms. Triplicate plots are shown. T100 % is the time to maximum absorbance, T0 % is the return of the optical denity to near-baseline. T50 % is (T100 % − T0 %)/2. The slope is the sharpest fall in optical density over time under the effect of exogenous tPA, effectively the reverse of the rate of clot formation in (**a**)

Thrombelastograph definitions as provided by manufacturer. Full details of the microplate assay indices are presented in Ref. [15]. See also Fig. 1

### Laboratory

Citrated venous blood was collected and analysed for indices of thrombogenesis and fibrinolysis using thrombelastography and by a microplate assay [12, 15]. The thrombelastograph assay uses whole blood, which is added to a small rotating cuvette alongside an activating solution of thromboplastin [16–19]. Formation of a clot is monitored by the resistance offered by the clot to the vibration of a probe. The microplate assay is conducted in plasma and consists of two parts [15]. Firstly, in a thrombogenesis assay, 25 μl plasma, 75 μl TRIS–NaCl buffer, and 50 μl thrombin are added to the wells of 96-well microtitre plate. Clot formation is followed for 30 min at 37 °C in a micro-titre plate reader by change in optical density (Fig. 1a). The key endpoints are lag time (time from addition of thrombin to the start of clot formation), rate of clot formation (change in optical density over time), and clot density (maximum change in optical density absorbance). Secondly, in the fibrinolysis assay, 75 μl plasma and 75 μl of a TRIS–NaCl/calcium/thrombin/tissue plasminogen activator cocktail are added to the wells of 96-well microtitre plate. Clot dissolution is followed for 30 min at 37 °C in a plate reader by change in optical density (Fig. 1b). The key endpoints are rate of clot dissolution, being the change in optical density over time on the downward slope of the right hand portion of the graph, and the time for 50 % fibrin clot lysis (T50).

Although the thrombelastograph delivers numerous indices, we determined the five that are most pertinent and which have similarities to those of the microplate assay [15]. The thrombelastograph R time and microplate assay lag time both reflect time taken for thrombogenesis to begin. The TEG K time marks time from the beginning of clot formation to when a fixed level of clot firmness is reached: there is no microplate assay equivalent of this index. The thrombelastograph ‘Angle’ and the microplate assay rate of clot formation both measure the rate clot growth. The thromboelastograph maximum amplitude and the microplate assay maximum optical density respectively assess maximum strength, stiffness or density of the developed clot, whilst the thrombelastograph LY30, and the microplate assays of rate of clot dissolution and the T50 (time for 50 % of the clot to be lysed) all provide indices of the ability of the formed clot to resist fibrinolysis. These indices are summarised in Table 1. Soluble P selectin was measured in citrated plasma by a commercial ELISA kit (R&D Systems, Abingdon, UK).

### Statistics

Our primary hypothesis was of a difference of 0.5 of a standard deviation in a test statistic between aspirin users and warfarin users. In view of our expectation to perform multiple analyses, we determined that p < 0.02 at a 1-beta of 0.90 would be significant, a power calculation that demands a sample size of 106 (that is, 53 patients in each of two groups). However, we determined to over-recruit by 20 % (that is, to 128), for additional confidence. The sample size also allows a multivariate logistic regression analysis of ten possible variables (that is, five indices from each laboratory method) versus the two categorical indices of aspirin or warfarin [21]. A sample size of 128 gives that 1-beta power of 0.8 to defend a correlation coefficient of 0.25 from types 1 and 2 error at 2p < 0.05 [21]. In each patient group n = 64 brings the power of 1-beta = 0. 8 and p < 0.05 for a correlation coefficient >0.35. We therefore recruited consecutive patients with heart failure, coronary artery disease or atrial fibrillation until this sample size was achieved in each group. Our secondary hypothesis was that there was a difference in a test statistic of 0.5 of a standard deviation between aspirin users with chronic heart failure and coronary artery disease, and between warfarin users with chronic heart failure and atrial fibrillation. To satisfy p < 0.05 and 1-beta = 0.8, a sample size of 64 is required in each case [21]. In this secondary hypothesis we determined we had insufficient power for a robust correlation analysis. We also determined to recruit a small number of patients with acute disease (that is, acute heart failure) to provide a perspective against chronic heart disease.

Data with a normal distribution are presented as mean and standard deviation and analysed by t test or analysis of variance. Data with a non-normal distribution are presented as median and inter-quartile range and analysed by the Mann-Whitney U test or the Kruskall–Wallis test. Where appropriate, data were correlated according to Pearson’s or Spearman’s method. Differences between three or more groups were sought by Tukey’s post-hoc test. Categorical indices were analysed by the Chi squared test. To determine which of the thrombelastograph or microplate assay indices were most closely associated with use of aspirin or warfarin, a multivariate logistic regression analysis was performed. All analyses were performed on Minitab release 16 (Minitab, Coventry, UK).

## Results

Table 2 shows clinical, demographic and routine laboratory data on the 181 patients with different forms of heart disease and on different medications. Table 3 shows data from patients with stable cardiovascular disease (i.e. excluding acute heart failure) on either of the two modes of antithrombotic therapy (antiplatelet vs anticoagulant). Of those on aspirin, 44 had coronary artery disease and 25 had chronic heart failure. Of those on warfarin, 32 had chronic heart failure and 60 had atrial fibrillation. Patients on warfarin were older than those on aspirin.

**Table 2:** Clinical, laboratory, demographic and therapeutic features of the participants

	Acute heart failure (n = 20)	Chronic heart failure (n = 57)	Atrial fibrillation (n = 60)	Coronary artery disease (n = 44)	p value
Age (years)	74.9 (10.5)	71.0 (12.1)	71.6 (8.9)	(68.7)	0.178
Sex (male/female)	17/3	41/16	42/18	29/21	0.140
BMI (kg/m^2^)	29.5 (7.0)	29.7 (5.8)	30.2 (8.0)	28.0 (5.5)	0.130
SBP (mmHg)	123 (17)	124 (14)	135 (19)	138 (18)	<0.001
DBP (mmHg)	71 (11)	73 (11)	77 (11)	74 (13)	0.130
eGFR (1.73/kg/m^2^)	49 (17)	57 (20)	61 (14)	68 (14)	0.001
Creatinine (μmol/L)	139 (80)	110 (37)	100 (31)	87 (22)	<0.001
CAD (n)	20	45	0	44	<0.001
AF (n)	10	36	60	0	<0.001
HF (n)	20	57	0	0	<0.001
Diabetes (n)	11	29	21	19	0.195
Antithrombotic therapy					
Antiplatelet (n)	10	25	0	44	<0.001
Warfarin (n)	10	32	60	0	<0.001
INR (in those on warfarin)	2.1 (0.3) (n = 7)	2.3 (0.7) (n = 29)	2.5 (0.7) (n = 47)	–	0.324
LMWH (n)	10	0	0	0	–
Other medications					
ACEI/ARB (n)	18	50	34	33	<0.001
CCB (n)	8	7	19	22	<0.001
Beta-blocker (n)	18	40	42	25	0.112
Aldo-anta (n)	10	23	5	2	<0.001
Loop diuretic (n)	20	42	18	11	<0.001
Statin (n)	18	43	37	37	0.019
Oral nitrates (n)	9	8	7	13	0.003
Hydralazine (n)	1	2	0	0	<0.001
Digoxin (n)	7	13	18	0	0.001

Data presented as mean and standard deviation, median and interquartile range, or number (%) of subjects. p value by analysis of variance or the Chi squared test

*CHF* chronic heart failure, *AHF* acute heart failure, *CAD* coronary artery disease, *AF* atrial fibrillation, *BMI* body mass index, *SBP* systolic blood pressure, *DBP* diastolic blood pressure, *eGFR* estimated glomerular filtration rate, *INR* international normalised ratio, *ACE/ARB* angiotensin converting enzyme inhibitor/angiotensin-receptor blocker, *CCB* calcium channel blocker, *Aldo-Anta* aldosterone antagonist

**Table 3:** Analysis according to anti-thrombotic therapy in patients with stable cardiovascular disease

Index	Aspirin (n = 69)	Warfarin (n = 92)	p value
Age (years)	68.1 (11.3)	72.5 (10.0)	0.011
Sex (male/female)	47/22	62/30	0.922
SBP (mmHg)	133 (17)	131 (19)	0.361
DBP (mmHg)	73 (13)	75 (11)	0.612
eGFR (1.73/kg/m^2^)	65 (18)	59 (15)	0.080
Creatinine (μmol/L)	96 (33)	103 (31)	0.163
BMI (kg/m^2^)	28.3 (5.3)	30.2 (7.4)	0.080
Diabetes (yes/no)	35/34	34/58	0.081
Thrombelastograph indices			
R time (min)	5.3 (4.4–6.5)	6.5 (5.5–8.7)	<0.001
K time (min)	1.4 (1.2–1.8)	1.8 (1.4–2.3)	0.001
Angle (°)	67.1 (7.5)	62.3 (8.7)	<0.001
Maximum amplitude (mm)	67.7 (4.3)	63.6 (10.7)	0.001
LY30 (%)	0.5 (0.1–1.4)	1.0 (0–0.7)	0.006
Microplate assay indices			
Lag time (min)	6.35 (2.58)	8.57 (3.38)	<0.001
Rate of clot formation (OD units/s)	25.2 (11.4)	17.7 (9.5)	<0.001
Maximum optical density (OD units)	0.35 (0.11)	0.40 (0.10)	0.012
Rate of clot dissolution (units/s)	42.1 (13.7)	47.5 (18.1)	0.043
T50 (min)	3.42 (0.77)	3.32 (0.85)	0.527

Data presented as mean and standard deviation, median and interquartile range, or number (%) of subjects. p value by t test, Mann–Whitney or Chi squared test

*SBP* systolic blood pressure, *DBP* diastolic blood pressure, *eGFR* estimated glomerular filtration rate, *LY30* lysis at 30 min, *T50* time for 50 % of the clot to lyse

In testing our primary hypothesis, all five thrombelastograph indices were markedly different between the anti-thrombotic groups by a factor of greater than 0.5 of a standard deviation (non-normal data log transformed). Those on warfarin had longer R and K times, a less steep Angle, a smaller maximum amplitude and higher LY30. Only four of the microplate assay indices differed between the two drug groups. As with the thrombelastograph, the lag time was longer and the rate of clot formation was slower, but the maximum optical density was greater in those on warfarin. Although the rate of clot dissolution was marginally slower on warfarin, the T50 was not altered. A recent INR was available on 83 patients being treated with warfarin (atrial fibrillation n = 47, acute heart failure n = 7, chronic heart failure n = 29). In this group, INR correlated weakly with the thrombelastograph R time (r = 0.25, p = 0.023), the Angle (r = −0.25, p = 0.024) and the LY30 (r = −0.27, p = 0.013). However, the sample size of 83 allows only for meaningful correlations with a coefficient that exceeds 0.3 [22], so that these relationships may be false positives. Nevertheless, the INR also correlated with the microplate assay index rate of clot formation (r = −0.34, p = 0.002), a result we believe is robust.

The multivariate regression analysis of data from patients with stable cardiovascular disease is shown in Table 4. Of the thrombelastograph indices, the R time, LY30 and maximum amplitude were all independently related to the nature of the antithrombotic (i.e. aspirin or warfarin). Similarly, of the microplate assay indices, the rate of clot formation, rate of clot dissolution, the lag time and maximum optical density were all independently related to the nature of the antithrombotic. Putting together those indices above in a third analysis, only microplate assay indices rate of clot formation, rate of clot dissolution and maximum optical density were independently related to the nature of the antithrombotic.

**Table 4:** Logistic regression of thrombelastograph and microplate assay indices versus anti-platelet or anticoagulant therapy

Predictor	Coefficient	SE Coef	Z	p	Odds ratio (95% CI)
Thromboelastograph indices alone					
R time	0.551343	0.158764	3.47	0.001	1.74 (1.27, 2.37)
LY30	−0.584913	0.235369	−2.49	0.013	0.56 (0.35, 0.88)
MA	−0.111962	0.047035	−2.38	0.017	0.89 (0.82, 0.98)
Angle	0.086552	0.062208	1.39	0.164	1.09 (0.97, 1.23)
K time	0.265357	0.682708	0.39	0.698	1.30 (0.34, 4.97)
Microplate assay indices alone					
RCF	−0.124420	0.028851	−4.31	<0.001	0.88 (0.83, 0.93)
RCD	0.053695	0.017064	3.15	0.002	1.06 (1.02, 1.09)
L time	0.004334	0.001414	3.07	0.002	1.00 (1.00, 1.01)
MOD	0.071971	0.025785	2.79	0.005	1.07 (1.02, 1.13)
T50	0.002828	0.004716	0.60	0.549	1.00 (0.99, 1.01)
Thrombelastograph and microplate assay indices					
MPA RCF	−0.109234	0.028386	−3.85	<0.001	0.90 (0.85, 0.95)
MPA RCD	0.059916	0.017783	3.37	0.001	1.06 (1.03, 1.10)
MPA MOD	0.056458	0.026578	2.12	0.034	1.06 (1.00, 1.11)
TEG LY30	−0.494109	0.253729	−1.95	0.051	0.61 (0.37, 1.00)
TEG R time	0.259616	0.134682	1.93	0.054	1.30 (1.00, 1.69)
MPA L time	0.002744	0.001634	1.68	0.093	1.00 (1.00, 1.01)
TEG MA	−0.067848	0.042077	−1.61	0.107	0.93 (0.86, 1.01)

*TEG* thrombelastograph, *MPA* microplate assay, *MA* maximum amplitude, *MOD* maximum optical density (both tests of clot integrity), *L time* lag time, *RCF* rate of clot formation, *RCD* rate of clot dissolution, *T50* time for 50 % of the clot to be lysed, *LY30* clot autolysis at 30 min (all three are measures of fibrinolysis)

Table 5 shows research indices in patients on aspirin. The thrombelastograph R time was longer in the acute heart failure group, but was no different between chronic heart failure and coronary artery disease. There were differences in all five microplate assays indices between the three groups, and most of these were in the acute heart failure groups compared to coronary artery disease and chronic heart failure. The maximum optical density was higher in acute heart failure than in both chronic heart failure and coronary artery disease. The rate of clot dissolution was slowest in acute heart failure than in both chronic heart failure and coronary artery disease, and the T50 % was longer in acute heart failure than in the other two groups. However, the lag time was markedly longer (by a mean of 90 s [29 %]) in chronic heart failure compared to coronary artery disease (p < 0.001). Similarly, the rate of clot formation was markedly slower (by a mean of 47.5 %) in chronic heart failure compared to coronary artery disease (p < 0.001).

**Table 5:** Research indices in patients on aspirin

Index	Acute heart failure (n = 10)	Chronic heart failure (n = 25)	Coronary artery disease (n = 44)	p value
Thromboelastograph indices				
R time (min)	6.9 (2.9)^a^	6.0 (1.7)	5.2 (1.3)	0.017
K time (min)	2.0 (1.1)	1.5 (0.6)	1.0 (0.6)	0.136
Angle (°)	62.0 (11.1)	66.8 (8.6)	67.3 (6.1)	0.161
Maximum amplitude (mm)	67.2 (9.6)	67.8 (4.2)	67.7 (4.4)	0.948
LY30 (%)	0.4 (0–1.32)	0.5 (0.05–1.55)	0.5 (0.07–1.4)	0.843
Microplate assay indices				
Lag time (min)	7.3 (4.5–15.4)	6.7 (5.7–16.7)	5.2 (4.5–6.0)^b^	<0.001
Rate of clot formation (OD units/s)	39.0 (7.0)^c^	20.0 (8.0)^c^	29.4 (12.0)^c^	<0.001
Maximum optical density (OD units)	0.55 (0.16)	0.33 (0.13)^d^	0.38 (0.08)^d^	<0.001
Rate of clot dissolution (OD units/s)	25.9 (3.4)	39.0 (13.7)^e^	44.5 (13.4)^e^	<0.001
T50 (min)	4.8 (0.55)	3.2 (0.9)^d^	3.6 (0.6)^d^	<0.001

Data presented as mean and standard deviation, median and interquartile range, or number (%) of subjects. p value by analysis of variance, between groups by Tukey’s post-hoc test

*LY30* lysis at 30 min, *T50* time for 50 % of the clot to dissolve

^a^Higher than in coronary artery disease

^b^Lower than in acute heart failure and chronic heart failure

^c^Difference significant between all three groups

^d^Lower than in acute heart failure

^e^Higher than in acute heart failure

Table 6 shows research indices in patients on anticoagulants. There were no significant differences in any thrombelastograph index between the three groups. However, maximum amplitude was marginally greater (by 7.4 %, p = 0.033) in chronic heart failure compared to atrial fibrillation, despite no difference in the INR (chronic heart failure 2.3 (0.7), atrial fibrillation 2.5 (0.7), p = 0.317). Regarding the microplate assay indices, the rate of clot formation and rate of clot dissolution were both slower, and the maximum optical density was higher in acute heart failure compared to both chronic heart failure and atrial fibrillation.

**Table 6:** Research indices in patients on warfarin

Index	Acute heart failure (n = 10)	Chronic heart failure (n = 32)	Atrial fibrillation (n = 60)	p value
Thrombelastograph indices				
R time (min)	6.1 (4.6–9.2)	6.6 (5.4–9.7)	6.5 (5.5–7.9)	0.564
K time (min)	1.3 (1.0–2.3)	1.8 (1.3–2.4)	1.8 (1.4–2.3)	0.240
Angle (°)	61.7 (17.2)	63.0 (9.6)	61.8 (8.2)	0.864
Maximum amplitude (mm)	65.4 (13.3)	66.6 (8.3)	62.0 (11.5)	0.144
LY30 (%)	0.15 (0–0.19)	0.2 (0–0.47)	0.1 (0–0.82)	0.917
Microplate asay indices				
Lag time (min)	8.2 (7.0–12.9)	7.4 (6.4–10.3)	7.9 (6.8–10.0)	0.553
Rate of clot formation (OD units/s)	37.6 (22.7–48.5)^a^	17.5 (11.3–26.4)	15.1 (10.8–20.2)	0.002
Maximum optical density (OD units)	0.53 (0.20)^a^	0.38 (0.11)	0.41 (0.10)	0.002
Rate of clot dissolution (OD units/s)	18.8 (8.5)^b^	49.4 (18.9)	46.4 (17.7)	<0.001
T50 (min)	3.7 (1.6)	3.35 (0.9)	3.3 (0.8)	0.553

Data presented as mean and standard deviation, median and interquartile range, or number (%) of subjects. p value by analysis of variance, between groups by Tukey’s post-hoc test

*LY30* lysis at 30 min, *T50* time for 50 % of the clot to dissolve

^a^Higher than in chronic heart failure and atrial fibrillation

^b^Lower in acute heart failure than in chronic heart failure and coronary artery disease

Soluble P selectin was 9.8 (7.6–11.2) ng/mL in acute heart failure, 9.0 (6.8–11.8) ng/mL in chronic heart failure, 9.6 (7.6–10.7) ng/mL in atrial fibrillation, and 8.6 (6.8–10.0) ng/mL in coronary artery disease (p = 0.31). Levels of soluble P selectin failed to correlate significantly with any index of renal function, thrombogenesis or fibrinolysis (data not shown). Excluding acute heart failure patients, levels were 8.4 (7.5–11.1) ng/mL in those patients on aspirin (including all those with coronary artery disease) and 9.6 (6.7–10.4) ng/mL in those patients (including all those with atrial fibrillation) on warfarin (p = 0.0175).

### Correlations

With a pre-specified correlation coefficient of 0.25, there were no meaningful relationships between any clinical or demographic index and any laboratory index. Significant correlations between haemostasis indices in the entire group are shown in Table 7. There were strong (correlation coefficient (r) >0.6) associations between the thromboelastograph R and K times, the R time and the Angle, and the K time with the Angle, but none between any microplate assay index. There were modest (r 0.35–0.59) associations between thromboelastograph indices maximum amplitude and K time, and maximum amplitude with the Angle, and between microplate assay indices rate of clot formation with maximum amplitude and of the rate of clot formation with T50. There were weak (r 0.25–0.34) but statistically significant associations between microplate assay indices lag time and rate of clot formation, and between the rate of clot dissolution and the T50. In seeking relationships between thrombelastograph indices and microplate assay indices, the microplate assay lag time correlated modestly with the thrombelastograph R time, the K time, and inversely with the Angle.

**Table 7:** Spearman correlates between haemostasis indices in 181 patients with cardiovascular disease

	R time	K time	Angle	Maximum amplitude
Thrombelastograph indices				
LY30	−0.13, 0.077	−0.20, 0.009	0.25, 0.001	−0.13, 0.097
Maximum amplitude	−0.24, 0.001	−0.50, <0.001	0.56, <0.001	
Angle	−0.64, <0.001	−0.87, <0.001		
K time	0.69, <0.001			

Microplate assay indices	Lag time	Rate of clot formation	Maximum optical density	Rate of clot formation
Microplate assay indices				
T50	−0.05, 0.525	0.35, <0.001	0.14, 0.073	−0.25, 0.001
Rate of clot dissolution	−0.16, 0.039	0.04, 0.607	0.08, 0.284	
Maximum optical density	0.13, 0.104	0.35, <0.001		
Rate of clot formation	−0.28, <0.001			

Microplate assay indices	Thrombelastograph indices				
	R time	K time	Angle	Maximum amplitude	LY30
Thromboelastograph indices vs microplate indices indices					
Lag time	0.47, <0.001	0.37, <0.001	−0.35, <0.001	−0.09, 0.257	−0.18, 0.02
Rate of clot formation	−0.23, 0.002	−0.22, 0.004	0.18, 0.021	0.17,0.027	0.19, 0.012
Maximum optical density	0.08, 0.315	−0.08, 0.334	0.09, 0.271	0.13, 0.085	−0.07, 0.371
Rate of clot dissolution	−0.10, 0.195	0.02, 0.807	0.02, 0.801	−0.08, 0.326	0.03, 0.652
T50	−0.06, 0.417	−0.02, 0.789	0.01, 0.850	0.14, 0.066	−0.03, 0.740

Data are Spearman correlation coefficient (r) and p value

## Discussion

In this study, our principal findings are that, although often assessing similar aspects of thrombogenesis and fibrinolysis, the thrombelastograph and microplate assay methods provide markedly different outcomes. For example, in the regression analysis, the two microplate assay indices of rate of clot formation and rate of clot dissolution were independent predictors of the nature of the anti-thrombotic, and in the aspirin users, all five microplate assay indices (but none of the thrombelastograph indices) varied between patient groups.

Thrombosis in cardiovascular disease still occurs despite best anti-thrombotic therapy, prompting research into pathophysiology and the effects of drugs on mechanisms of thrombosis. Although valuable, existing laboratory measures for assessing haemostasis, including the thrombelastograph, have limitations [9, 11, 19, 23–25]. The microplate assay has some features in common with the thrombelastograph, and has other features, such as the assessment of fibrinolysis initiated by exogenous tPA [15]. However, there are several alternative assays that also assess fibrin clot formation and fibrinolysis, some of which are performed in a micro-titre plate [13, 14, 26–28]. For example, that of Talens et al. [27] produces a tPA-mediated fibrinolysis time of over 80 min, whilst Zabczyk et al. [28] reported how warfarin rapidly influences indices of clot integrity in patients with atrial fibrillation. Soluble P selectin is known to be increased in all major cardiovascular disease [29–31], and in our hands, do not differ between those with atrial fibrillation, coronary artery disease or heart failure.

### Methodological aspects

In our study of 181 patients with various cardiovascular diseases and on different therapies, three thrombelastograph indices (R time, K time and Angle) inter-correlated strongly suggesting they measure similar aspects of thrombogenesis, whilst two pairs of indices (maximum amplitude with K time and Angle) correlated modestly, suggesting they too share certain features of haemostasis. In the microplate assay, the rate of clot formation correlated with the maximum optical density (which is plausible as they respectively mark the rate of clot formation and its density once formed). However, the same strength of correlation between the rate of clot formation and the T50 is less easy to explain but may reflect clot structure. The thrombelastograph R time and microplate assay lag time correlated modestly, suggesting they too share common features, these being those related to clot formation.

Unsurprisingly, all indices related to clot formation and integrity were different in those on aspirin (and so an undisturbed coagulation pathway) compared to warfarin (with a disturbed coagulation pathway). The finding that soluble P selectin is lower in those on aspirin confirms other data [32, 33] and the effect of this drug on platelets. Delayed initiation of thrombogenesis in warfarin use was apparent with prolonged TEG R and K times and the microplate lag time. Similarly, slower clot growth was demonstrated by altered thrombelastograph Angle and the microplate assay rate of clot formation. Use of warfarin provided a less robust whole blood clot (thrombelastograph maximum amplitude), but the higher microplate assay maximum optical density result on warfarin implies a denser fibrin clot than in those using aspirin. The reasons for this are unclear but are likely to relate to an effect of blood cells on the thrombelastograph assay. Of the 83 patients taking warfarin, the INR correlated most strongly with the microplate assay rate of clot formation, but less so with the thrombelastograph R time and the Angle, all of which seems likely as warfarin impairs clot formation. To some extent this latter result is counter to the report by Franchi et al. [19] of 100 patients on warfarin, who found the correlation between the INR and thrombelastograph to be barely not significant (r = 0.19, p = 0.06), which may be due to difference in patients between the two studies. Franchi et al. [19] also failed to significantly correlate the INR with other thrombelastograph indices, and taken together, this may because of the combined effects of all three types of blood cells on clot formation and dissolution (such as the release of platelet-stimulating ADP by red cells, and expression of pro-coagulant tissue factor by monocytes). Indeed, one way of improving the thrombelastograph is to modify the process by adding exogenous tissue factor to the kaolin to accelerate the process of clot formation [19]. However, the correlation we found between the INR and TEG LY30 index may be explained as a weaker clot (marked by a high INR) being more susceptible to autolysis.

Although all five thrombelastograph indices were different between the two antithrombotic classes, in multivariate analysis only the R time remained a strong significant independent predictor. This can be accounted for by the many strong or moderate correlations between certain of these five indices (Table 7). This is in contrast to the four microplate assay indices, which were all retained in multivariate analysis, implying each offer a different independent aspect of clot formation. In a combined analysis, the two microplate assay indices of rate of clot formation and rate of clot dissolution both remained strongly linked to the use of warfarin or aspirin, whilst the thrombelastograph R time was eliminated.

### Clinical aspects: aspirin users

Seventy-nine patients were taking aspirin, and are therefore expected to have an ‘intact’ coagulation pathway. All five microplate assay indices were markedly (p < 0.001) different between the three patient groups, but only one thrombelastograph index (the R time) was marginally (p = 0.017) different between the three groups. However, a great deal of this variability may be due to the gross abnormalities in the acute heart failure patients, all of whom were in-patients and therefore in need of enhanced care, and of whom half were being treated with a low molecular weight heparin to prevent inpatient venous thromboembolism. In comparing only those with chronic, stable disease (i.e. chronic heart failure v coronary artery disease), thrombogenesis in plasma (as defined by the microplate assay lag time) was more rapid in coronary artery disease than in chronic heart failure. This may be because, in these patients, the pathophysiology of their coronary artery disease is more thrombogenic than those with chronic heart failure, none of whom had a history of, or overt, coronary artery disease.

A further possible effect on these indices is the effect of other classes of pharmaceuticals such as nitrates and statins. In addition, coronary artery disease patients had a more rapid rate of clot formation than the chronic heart failure patients. This also imply that patients with coronary artery disease on aspirin are at increased risk of thrombosis compared to chronic heart failure patients on aspirin, and perhaps soluble platelet products such as beta-thromboglobulin may take part in plasma thrombogenesis [34, 35]. The difference in rate of clot formation in the chronic heart failure group compared to the coronary artery group suggests it may have potential as a marker of thrombogenicity in aspirin users, as does the INR in warfarin users.

### Clinical aspects: warfarin users

One hundred and twelve patients were taking warfarin, and will therefore have a ‘disrupted’ coagulation pathway. Only the microplate assay indices rate of clot formation, maximum optical density and rate of clot dissolution were different between the groups. Once more, a great deal of this variability may be due to the gross abnormalities in haemostasis among the acute heart failure patients. It suggests that acute heart failure patients possess greater thrombotic potential (faster rate of clot formation), and form thicker clots (greater maximum optical density), which are more resistant to exogenous tPA-induced fibrinolysis (with slower rate of clot dissolution). However, we speculate that a great deal of these differences are due to low molecular weight heparin therapy in 50 % of the acute heart failure patients, reflecting the data of Incampo et al. [14] who, using a thrombelastograph and a turbidimetry assay, showed the effects of this anticoagulant on indices of haemostasis. In comparing only those with chronic, stable disease (i.e. chronic heart failure v atrial fibrillation), those with chronic heart failure generated a slightly more robust clot (higher maximum amplitude) than those atrial fibrillation patients. It may also be the case that the thrombelastograph and/or the microplate assay could be used, if necessary, to assess thrombogenicity in the absence of an INR. The lack of strong correlations between the INR and thrombelastograph indices supports the contention of Franchi et al. [19] that the thrombelastograph is not a useful tool to evaluate VKA anticoagulant effect, compared with standard INR measurements, although a modified rapid technique shows promise.

### Thrombelastography and the microplate assay compared

As the thrombelastograph operates on whole blood and the microplate assay uses plasma, the two methods are fundamentally different. Indeed, thrombelastograph indices can be directly influenced by platelets [36, 37], and may be useful in assessing the activity of NOACs [17, 38]. Nevertheless, both techniques measure thrombogenesis, clot integrity, and fibrinolysis. Several of the thrombelastograph indices (Angle, R time, K time) provide similar information (correlations coefficient >0.56), whereas all of the microplate assays indices are (by and large) independent of each other (correlations coefficients <0.35). Although all of the thrombelastograph indices, but only four of the microplate assay indices were different in the presence of disrupted coagulation, in multivariate analysis, only the microplate rate of clot formation and rate of clot dissolution indices were independent predictors of the use of warfarin, suggesting they may have real value in a clinical setting.

The microplate lag time and the rate of clot formation also show promise as clinical tools, being more adverse in coronary artery disease than in chronic heart failure. This is surprising as the primary reason for a chronic heart failure patient being on aspirin is because of underlying arterial disease, as is the case in 80 % of our chronic heart failure patients. This point is also pertinent as patients with CHF may be treated with anti-platelets or with warfarin [7], and so may be relevant in clinical decision-making.

### Limitations

We acknowledge several limitations, the principal methodological one being that of whole blood versus plasma, in that the latter does not allow discussion of the effects of cells. Conversely, differences in the certain indices between two methods may well be due to the cellular components of the blood. Centrifugation was at a ‘standard’ speed and duration (3000 rpm, 20 min) so we cannot exclude the possibility of the presence of small platelets or procoagulant platelet microparticles in the plasma [39]. The thrombelastograph is far easier to operate: the microplate assay demands technical skill in reagent preparation and sample processing, although overall the MPA is far more economical than the thrombelastograph. From the clinical perspective it is distantly possible some indices may be influenced by other drugs such as nitrates and statins. Although renal function differed between patient groups, this did not associate with any thrombelastograph or microplate assay, despite suggestions that this may be the case [18, 40]. Finally, we acknowledge that is important to note that, as shown for other assays assessing effects of antiplatelet agents which have differences in technical aspects, even for these assays used in this study, methodological variations may explain differences in pharmacodynamics.

## Conclusions

As with other related methods [14, 26–28], such as the rapid-thrombelastograph [19], the microplate assay shows promise as an alternative tool for dissecting thrombogenesis and fibrinolysis in cardiovascular disease, and the impact of anti-thrombotic therapy. Large prospective studies in coronary artery disease, heart failure and atrial fibrillation are required to ascertain the clinical value of these mechanistic biomarkers for determining the risk of major cardiovascular events and thromboembolism in heart disease.
